# Targeting ER stress in skeletal muscle through physical activity: a strategy for combating neurodegeneration-associated muscle decline

**DOI:** 10.3389/fnmol.2025.1639114

**Published:** 2025-10-21

**Authors:** Zhanguo Su, Lijuan Xiang

**Affiliations:** ^1^Faculty of Physical Education, Huainan Normal University, Huainan, Anhui, China; ^2^Chongqing Preschool Education College, Chongqing, China

**Keywords:** exercise, ER stress, muscle function, neurodegenerative diseases, unfolded protein response

## Abstract

The pathophysiology of neurodegenerative diseases is largely driven by ER stress, contributing to cellular dysfunction and inflammation. Chronic ER stress in skeletal muscle is associated with a deterioration in muscle function, particularly in diseases such as ALS, PD, and AD, which are often accompanied by muscle wasting and weakness. ER stress triggers the UPR, a cellular process designed to restore protein homeostasis, but prolonged or unresolved stress can lead to muscle degeneration. Recent studies indicate that exercise may modulate ER stress, thereby improving muscle health through the enhancement of the adaptive UPR, reducing protein misfolding, and promoting cellular repair mechanisms. This review examines the influence of exercise on the modulation of ER stress in muscle cells, with a particular focus on how physical activity influences key pathways contributed to mitochondrial function, protein folding, and quality control. We discuss how exercise-induced adaptations, including the activation of stress-resilience pathways, antioxidant responses, and autophagy, can help mitigate the negative effects of ER stress in muscle cells. Moreover, we examine the potential therapeutic implications of exercise in neurodegenerative diseases, where it may improve muscle function, reduce muscle wasting, and alleviate symptoms associated with ER stress. By integrating findings from neurobiology, muscle physiology, and cellular stress responses, this article highlights the therapeutic potential of exercise as a strategy to modulate ER stress and improve muscle function in neurodegenerative diseases.

## 1 Introduction

A growing worldwide health issue, neurodegenerative diseases affect millions and place great strain on people, families, and medical systems (Marques-Aleixo et al., 2021). These conditions are characterized by the progressive loss of neuronal structure and function, resulting in cognitive decline, it is increasingly recognized that their impact extends beyond the CNS to affect motor abilities and muscle health (Marques-Aleixo et al., 2021; de Lima et al., 2024; D’Angelo and Bresolin, 2006). Peripheral manifestations, particularly in skeletal muscle, are significant and involve substantially in the overall disease progression, reducing the quality of life and increasing morbidity in affected every person (Deldicque, 2013; Estébanez et al., 2018). This systemic impact suggests that therapeutic strategies should not solely focus on the neurological aspects but also consider the peripheral components of these debilitating conditions.

ER stress is a critical cellular mechanism associated with the pathogenesis of various diseases, including neurodegenerative disorders (Afroze and Kumar, 2019). The ER, a vital organelle within cells, is essential for protein synthesis, folding, modification, and trafficking. Maintaining balance within the ER, is crucial for optimal cellular function, especially in dynamic tissues such as skeletal muscle (Deldicque, 2013; Bohnert et al., 2018). Skeletal muscle, constituting a significant portion of body mass, is critical for locomotion, posture maintenance, breathing, and whole-body metabolism (Deldicque, 2013; Argilés et al., 2016). Its ability to function correctly is paramount for overall health and the capacity to perform daily activities.

Exercise has emerged as a promising non-pharmacological intervention in the quest for effective treatments for neurodegenerative diseases, with the potential to modulate various cellular stress pathways, including ER stress (Santiago and Potashkin, 2023). As a systemic intervention, exercise holds the potential to address both the neurological and muscular components of these diseases by influencing key shared cellular stress mechanisms like ER stress. The paper presents a review of the role of exercise in modulating ER stress and its impact on muscle function in PD, AD, Huntington’s disease, and ALS. By synthesizing current research findings, this report seeks to illuminate potential therapeutic benefits and identify avenues for future interventions.

## 2 Understanding endoplasmic reticulum stress and muscle function

The Endoplasmic Reticulum (ER) is a dynamic network of membranes within eukaryotic cells, fulfilling diverse and critical functions. In muscle cells, this organelle exists in a specialized form known as the sarcoplasmic reticulum (SR) (Deldicque, 2013; Zhang et al., 2021). The ER/SR is central to muscle function, regulating both the calcium homeostasis required for contraction and the protein quality control (synthesis, folding, and trafficking) essential for muscle maintenance (Deldicque, 2013; Delmotte and Sieck, 2015). Due to this central role in proteostasis, the muscle ER/SR is highly susceptible to stress. For clarity and consistency with the broader literature on the unfolded protein response, this review will use the term ‘ER stress’ throughout, with the understanding that in the context of muscle, this refers to stress within the SR.

ER stress arises when the protein folding capacity of this organelle is exceeded due to the accumulation of misfolded or unfolded proteins within its lumen (Deldicque, 2013; Gregersen and Bross, 2010). This imbalance can arise from a variety of pathological and physiological insults particularly relevant to skeletal muscle, including oxidative stress, hypoxia, nutrient excess (e.g., high-fat diet-induced lipotoxicity), systemic inflammation, calcium dysregulation, muscle disuse or denervation, and the accumulation of misfolded proteins associated with aging and specific disease states (Afroze and Kumar, 2019; Rotariu et al., 2022). The presence of these stressors impairs the ER’s capacity to correctly fold and process proteins, thereby activating a highly conserved intracellular signaling network referred to as the UPR (Afroze and Kumar, 2019; Schröder and Kaufman, 2005).

The UPR is a complex adaptive mechanism that is designed to restore homeostasis in the ER (Afroze and Kumar, 2019; Coleman and Haller, 2019). It does this by modulating protein synthesis to reduce ER load, upregulating ER chaperone expression to aid protein folding, and increasing misfolded protein degradation through ERAD pathways (Afroze and Kumar, 2019; Nishikawa et al., 2005). PERK, IRE1α, and ATF6 are the main ER transmembrane sensors that mediate the UPR (Deldicque, 2013; Siwecka et al., 2019; Fang et al., 2022). These sensors activate specific downstream signaling pathways that together function to mitigate ER stress.

While the UPR is initially a protective response, chronic ER stress leads to detrimental consequences for skeletal muscle. Extended activation of the UPR transitions from an adaptive to a maladaptive response, directly promoting muscle pathology through several mechanisms (Deldicque, 2013; Cirone, 2021). First, prolonged activation of the PERK and IRE1α pathways can trigger apoptosis in myocytes, notably through the upregulation of the pro-apoptotic transcription factor CHOP (Tabas and Ron, 2011; Kadowaki and Nishitoh, 2013). This leads to a loss of muscle fibers and contributes directly to atrophy. Second, the UPR can initiate potent inflammatory pathways, such as NF-κB signaling, leading to the production of cytokines that impair insulin sensitivity and promote protein degradation (Saaoud et al., 2024; Hotamisligil, 2010). Third, persistent phosphorylation of eIF2α by PERK, intended to reduce protein synthesis, can chronically suppress the translation of essential contractile proteins, further exacerbating muscle wasting and weakness. In the context of muscle, this combination of apoptosis, inflammation, and impaired protein synthesis manifests as muscle dysfunction, atrophy (loss of muscle mass), and weakness, impacting overall physical capacity (Lin et al., 2025; Gordon et al., 2013). It is critical to explicitly differentiate between an adaptive (physiological) UPR and a maladaptive (pathological) UPR. The adaptive response, often triggered by moderate exercise, is transient and aims to restore homeostasis. Its hallmarks are the temporary activation of the IRE1α and ATF6 pathways to increase the ER’s protein-folding capacity by upregulating chaperones like BiP and enhancing ERAD (Kaufman et al., 2010; Wu et al., 2011). In contrast, a maladaptive response occurs when the stress is too severe or prolonged, overwhelming the adaptive capacity. This state is characterized by the sustained activation of the PERK pathway, leading to chronic translational repression, and the strong upregulation of the pro-apoptotic transcription factor CHOP, which ultimately triggers inflammation and cell death (Sano and Reed, 2013; Yang et al., 2017). The goal of therapeutic exercise is to consistently activate the adaptive UPR without tipping the balance into a maladaptive state.

**Figure 1:**
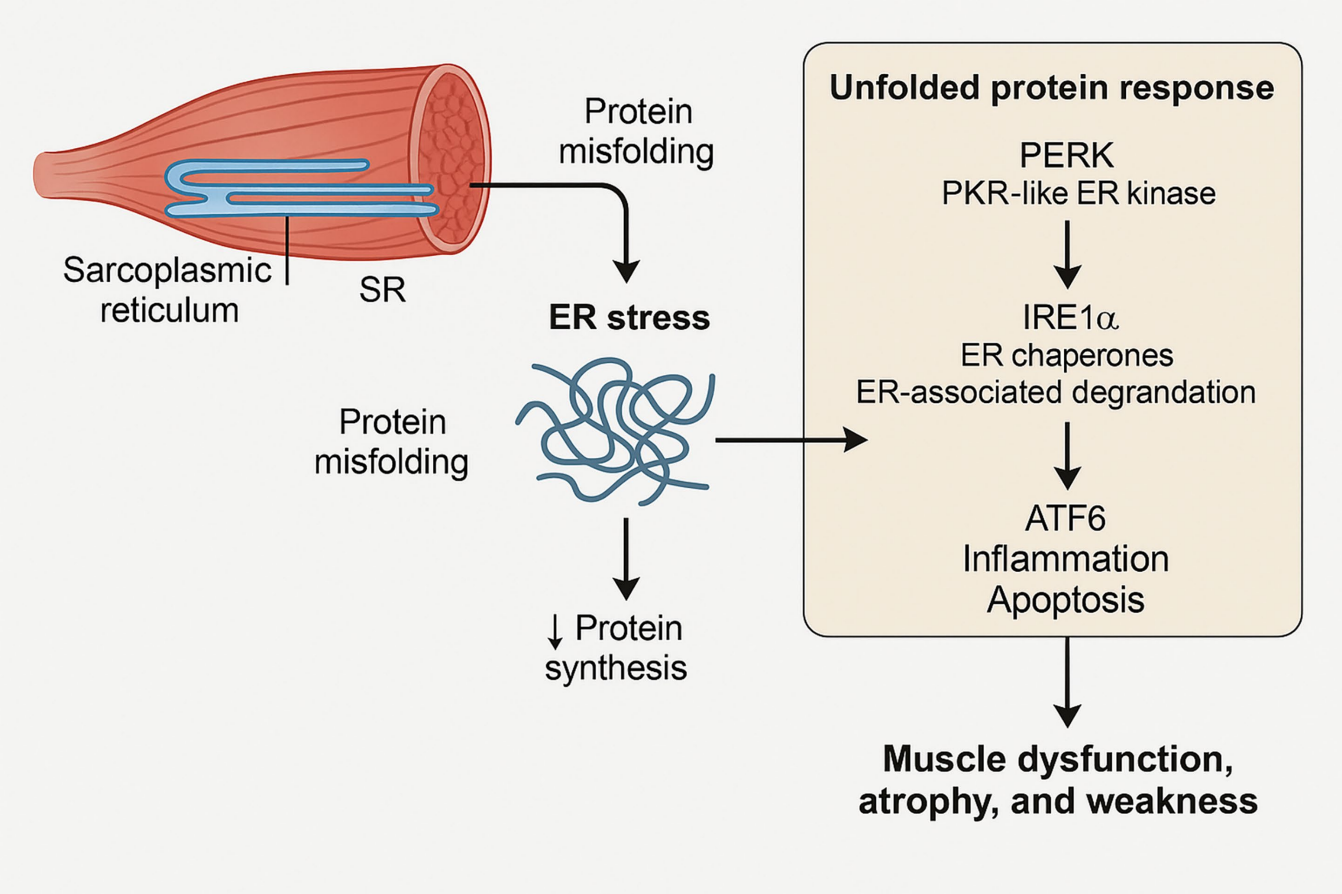
UPR and ER stress in muscle health. Sarcoplasmic reticulum (SR) controls protein quality and calcium homeostasis in muscle cells. SR protein misfolding causes ER stress and UPR activation under stress conditions like oxidative stress or inflammation. The UPR functions through three pathways: PERK (reduces protein synthesis), IRE1α (enhances chaperone expression and ER degradation), and ATF6 (induces inflammatory and apoptotic responses). While initially protective, prolonged UPR activation causes muscle dysfunction, atrophy, and weakness. This figure provides a schematic summary of the canonical UPR pathways as they relate to skeletal muscle pathology, based on the literature reviewed herein.

Therefore, maintaining a delicate balance in ER function is paramount for overall muscle health. The UPR’s duality, acting as both a protector and a potential instigator of damage under prolonged stress, highlights the complexity of this cellular response (Figure 1).

## 3 The role of ER stress in specific neurodegenerative diseases and muscle dysfunction

**Figure 2:**
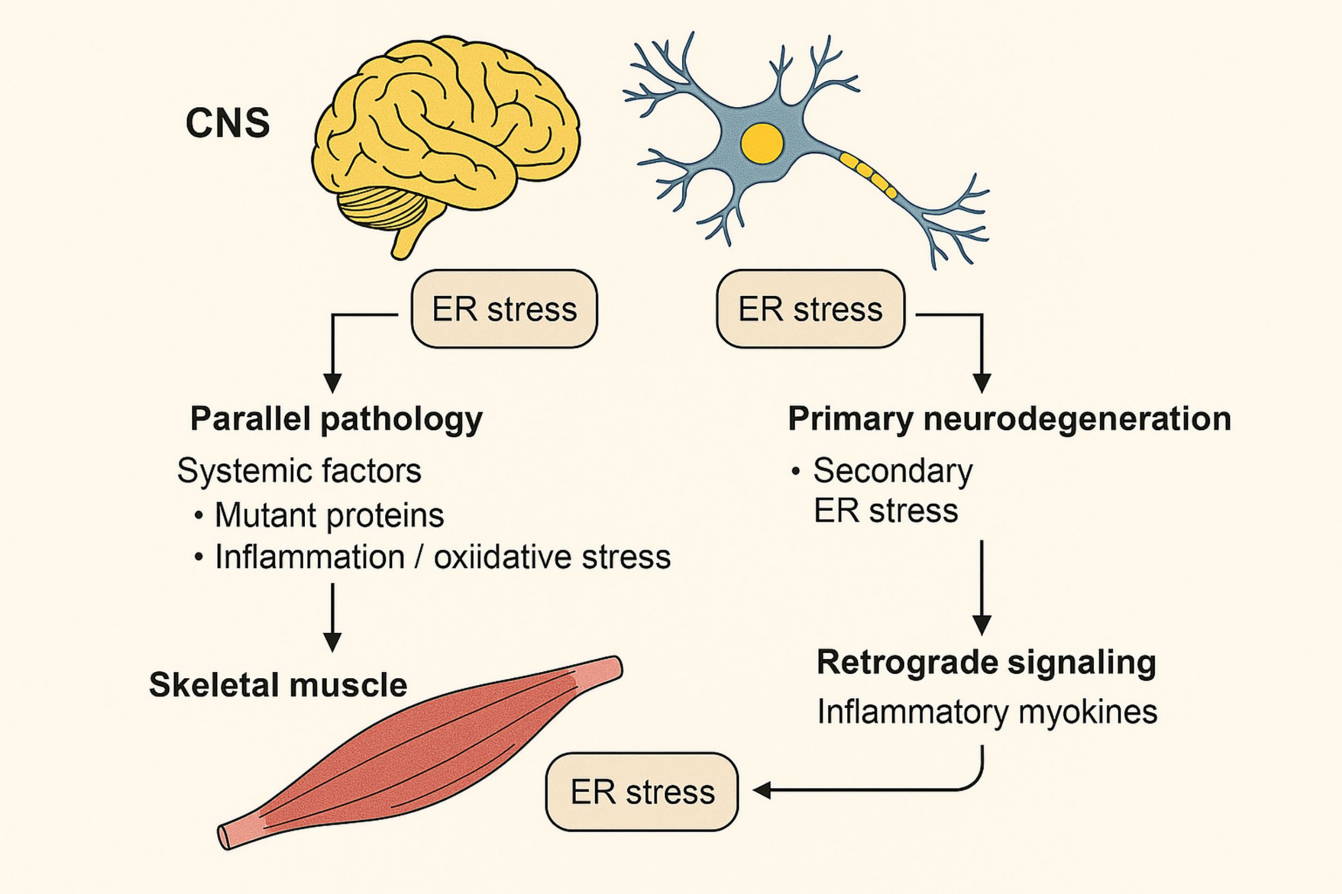
Potential links between ER stress in the central nervous system (CNS) and skeletal muscle in neurodegenerative diseases. Three major mechanisms may explain the correlation between CNS and muscle ER stress: (i) Parallel pathology, where systemic factors such as mutant proteins, inflammation, and oxidative stress induce ER stress independently in both neurons and muscle; (ii) Indirect consequential link, where primary neurodegeneration leads to muscle denervation, disuse atrophy, and secondary ER stress; and (iii) Retrograde signaling, where atrophying muscle releases inflammatory myokines that can cross the blood–brain barrier and exacerbate neuroinflammation and neuronal ER stress. Together, these processes create a bidirectional interaction contributing to disease progression.

A critical question in the pathophysiology of neurodegenerative diseases is how ER stress in the central nervous system (CNS) correlates with the ER stress observed in peripheral skeletal muscle. The connection is likely multifactorial rather than a single direct cause. One major mechanism is a parallel pathology driven by systemic factors; for instance, the widespread expression of mutant proteins (like mHTT in Huntington’s disease) or the presence of systemic inflammation and oxidative stress can independently induce ER stress in both neurons and muscle cells (Joshi et al., 2025; Kumar and Ratan, 2016; Cisbani and Cicchetti, 2012). A second mechanism involves an indirect, consequential link, where primary neurodegeneration in the CNS leads to secondary ER stress in the muscle. This can occur through processes like denervation, disuse atrophy, or altered neural signaling, all of which are potent triggers for muscle ER stress (Yadav and Dabur, 2024; Castelli et al., 2025). Finally, there may be a retrograde signaling component, where stressed or atrophying muscle releases inflammatory myokines that can cross the blood–brain barrier, potentially exacerbating neuroinflammation and neuronal ER stress (Rai and Demontis, 2022; Lee et al., 2021). Understanding these potential links is crucial for developing holistic therapeutic strategies that address both central and peripheral manifestations of these diseases (Figure 2).

### 3.1 Parkinson’s disease (PD)

In Parkinson’s Disease (PD), ER stress is well-established as a pivotal element in the pathogenesis within the central nervous system (Baek et al., 2019). The primary neuropathology involves the loss of dopaminergic neurons, where the accumulation of misfolded alpha-synuclein directly triggers the UPR, compromises ER function, and contributes to a toxic cycle of protein misfolding and neuronal death (Li, 2023). The vast majority of mechanistic studies on ER stress in PD have been conducted in this neuronal context.

The focus on CNS pathology often overshadows peripheral manifestations; however, PD motor symptoms like muscle rigidity and bradykinesia point to significant muscle involvement (Kong et al., 2025; Palakurthi and Burugupally, 2019). The link to ER stress in muscle is twofold. First, it can be inferred as an indirect consequence of CNS pathology; impaired neuronal signaling and motor control can lead to disuse or altered muscle activity, which are known stressors (Bernard-Marissal et al., 2015; Paschen and Doutheil, 1999). Second, and more directly, emerging evidence now suggests a primary ER stress response within muscle tissue itself, although this research is less extensive than the CNS-focused work. Studies have begun to identify UPR activation in the muscle of PD models, suggesting that the cellular stress response is not confined to the brain (Deldicque, 2013; Zhang et al., 2015; Mercado et al., 2016). This distinction is critical: muscle dysfunction may not just be a downstream effect of neurodegeneration but also a parallel pathology.

### 3.2 Alzheimer’s disease (AD)

While the role of ER stress in the neurons of the AD brain is well-documented, its involvement in peripheral skeletal muscle is an area of active investigation rather than established fact. Patients with AD frequently experience significant physical decline, including muscle weakness and sarcopenia, that cannot be fully explained by cognitive impairment alone (Endres and Reinhardt, 2013; Uddin et al., 2020; Ekundayo et al., 2024). The link to ER stress in muscle is therefore largely inferred from the systemic nature of the disease. It is hypothesized that systemic factors present in AD, such as chronic inflammation or circulating amyloid-*β* oligomers, could induce a parallel ER stress response in muscle tissue, thereby contributing to the observed frailty (Deldicque, 2013; Burtscher et al., 2021). It is crucial to differentiate this hypothesis from the direct evidence seen in the CNS, where amyloid-*β* and tau aggregates are known to trigger the UPR (Li et al., 2015; Ajoolabady et al., 2022). Thus, while plausible, the contribution of muscle-specific ER stress to AD pathology requires more direct experimental validation.

### 3.3 Huntington’s disease (HD)

An expanded CAG repeat in the huntingtin gene produces a mHTT protein with an abnormally long polyglutamine stretch, causing HD, a progressive neurodegenerative disorder (Maity et al., 2022; Gil and Rego, 2008). The accumulation of this misfolded mHTT protein within neurons induces significant ER stress and disrupts ER homeostasis (Maity et al., 2022). ER stress contributes to neuronal dysfunction, ultimately resulting in the neuronal death observed in HD.

HD is characterized by a triad of symptoms: motor impairments, cognitive decline, and psychiatric disturbances. The motor impairments—including chorea, dystonia, and rigidity—are accompanied by significant muscle atrophy and weakness that contribute substantially to functional decline (Goh et al., 2018; Mehanna and Jankovic, 2024). While direct evidence for ER stress in the muscle of HD patients is still emerging, strong indirect evidence links the pathology to this pathway. The mutant huntingtin (mHTT) protein is expressed universally, including in muscle tissue, where it forms aggregates (Jodeiri Farshbaf and Ghaedi, 2017; Barron et al., 2021). The presence of these aggregates can directly overwhelm the muscle’s protein quality control systems, such as the ER-associated degradation (ERAD) pathway, a classic trigger for the UPR. Furthermore, muscle biopsies from HD models show significant mitochondrial dysfunction and impaired autophagy, pathologies mechanistically linked to chronic ER stress. A key element of this pathology is the disruption of ER-mitochondrial crosstalk; ER stress alters calcium signaling between the two organelles, which in turn cripples mitochondrial bioenergetics and promotes cell death in muscle fibers (Maity et al., 2022; Vidal et al., 2011; Volgyi et al., 2015). Therefore, it is highly plausible that mHTT accumulation in muscle fosters a state of chronic ER stress that drives intrinsic muscle wasting (Afroze and Kumar, 2019; Hassab et al., 2023). Given this systemic pathology, investigating ER stress in peripheral tissues like muscle could uncover vital therapeutic targets beyond the brain. Strategies aimed at alleviating ER stress or improving ERAD function hold promise for improving both neurological and muscular symptoms in this devastating disease (Maity et al., 2022; Lontay et al., 2020).

### 3.4 Amyotrophic lateral sclerosis (ALS)

ALS or Lou Gehrig’s disease, is a neurodegenerative disorder that is characterized by the selective degeneration of motor neurons in the brain and spinal cord. It is a condition that progresses rapidly and ultimately results in death (Duranti and Villa, 2023; Loeffler et al., 2016). This neuronal degeneration results in progressive muscle atrophy, weakness, and ultimately paralysis, culminating in respiratory failure (Tiryaki and Horak, 2014; Wijesekera and Leigh, 2009). ER stress affects motor neurons and skeletal muscle tissue, contributing to ALS (Zhao et al., 2022; Jeon et al., 2023).

In contrast to other neurodegenerative conditions where muscle pathology is often considered secondary or inferred from CNS dysfunction, ALS presents compelling direct evidence for ER stress as a primary event in skeletal muscle tissue (Loeffler et al., 2016).

In ALS, the buildup of misfolded proteins like mutant superoxide dismutase 1 (SOD1) and TDP-43 is a key pathological driver that directly induces ER stress in muscle. For example, mutant SOD1 is known to accumulate within the ER and on the outer mitochondrial membrane of muscle cells, where it physically disrupts protein folding machinery and impairs calcium homeostasis, leading to a robust UPR activation (Nagaraju et al., 2005; de Mena et al., 2021). Importantly, research in ALS animal models shows that markers of ER stress (e.g., CHOP, BiP) are upregulated in skeletal muscle during pre-symptomatic stages, before significant denervation occurs (Nagaraju et al., 2005; Chen et al., 2015). This early activation provides strong evidence that muscle is not a passive victim of motor neuron loss but rather a primary site of pathology, with intrinsic ER stress contributing directly to atrophy by disrupting protein translation and activating pro-apoptotic pathways (Nagaraju et al., 2005; Jesse et al., 2017; Galbiati et al., 2014). This muscle-centric pathology helps explain why the role of exercise in ALS is uniquely complex and controversial (de Almeida et al., 2012; Tsitkanou et al., 2019). While moderate activity may be beneficial, strenuous exercise is hypothesized to be detrimental by overwhelming already vulnerable motor neurons. This could occur through several mechanisms, including increased metabolic load, excitotoxicity at the neuromuscular junction, and heightened oxidative stress that the compromised neuron cannot buffer (Duranti and Villa, 2023; Siciliano et al., 2020; Dishman et al., 2006; Sheikh and Vissing, 2019; Dobrowolny et al., 2021). This potential for harm underscores that any therapeutic strategy, including exercise, must account for the primary pathology. Therefore, the strong evidence for muscle-intrinsic ER stress in ALS not only highlights a therapeutic target but also provides a rationale for extreme caution with exercise prescription, demanding highly individualized and carefully monitored programs (Table 1).

**Table 1:** Summary of ER stress markers in neurodegenerative diseases.

Disease	Marker	Tissue	Change	References
Parkinson’s disease	GRP78/BiP	Brain (SNpc)	Increased	Kong et al. (2025)
Parkinson’s disease	CHOP	Brain	Increased	Kong et al. (2025)
Parkinson’s disease	XBP-1	Brain	Increased	Kong et al. (2025)
Parkinson’s disease	p-PERK	Brain (SNpc)	Observed	Wang et al. (2023)
Parkinson’s disease	p-eIF2α	Brain (SNpc)	Observed	Wang et al. (2023)
Parkinson’s disease	p-IRE1α	Brain (SNpc)	Observed	Wang et al. (2023)
Alzheimer’s disease	PERK	Temporal cortex	Increased	Li et al. (2015)
Alzheimer’s disease	eIF2α	Temporal cortex	Increased	Li et al. (2015)
Alzheimer’s disease	CHOP	Temporal cortex	Increased	Li et al. (2015)
Alzheimer’s disease	BiP	Temporal cortex	Increased	Li et al. (2015)
Alzheimer’s disease	XBP1 (spliced)	Temporal cortex & hippocampus	Increased	Li et al. (2015)
Huntington’s disease	Multiple UPR markers	Brain	Activated	Li (2023)
ALS	PERK	Skeletal muscle	Upregulated	Chen et al. (2015)
ALS	IRE1α	Skeletal muscle	Upregulated	Chen et al. (2015)
ALS	p-eIF2α	Skeletal muscle	Increased	Chen et al. (2015)
ALS	CHOP	Skeletal muscle	Upregulated	Chen et al. (2015)
ALS	Grp78/BiP	Skeletal muscle	Increased	Chen et al. (2015)

## 4 Exercise as a modulator of ER stress in skeletal muscle

### 4.1 Acute vs. chronic exercise

Physical exercise has a multifaceted impact on ER stress in skeletal muscle, with notable differences observed between acute and chronic exercise protocols (Petriz et al., 2017; Marafon et al., 2022). Acute exercise, including endurance and resistance activities, can trigger ER stress and activate the unfolded protein response in skeletal muscle (Bohnert et al., 2018; Egan and Sharples, 2023). This initial stress response is likely due to the increased protein synthesis demands and fluctuations in calcium levels that occur during muscle contraction (Berchtold et al., 2000; Kameyama and Etlinger, 1979). ER stress markers like BiP, CHOP, GRP94, and phosphorylated eIF2α have been found to increase in skeletal muscle after a single bout of intense exercise (Afroze and Kumar, 2019; Gallot and Bohnert, 2021; Khadir et al., 2016; Marafon, 2022). This suggests that the UPR activation following acute exercise could be an adaptive mechanism, preparing the muscle for future challenges.

Chronic regular moderate-intensity exercise induces adaptations that reduce the responses of genes and proteins associated with ER stress in skeletal muscle (Li et al., 2019; Korkmaz et al., 2023). This suggests that consistent exercise training can enhance the muscle’s ability to cope with cellular stress, potentially protecting it against subsequent stressors. However, it is crucial to note that excessive exercise intensity and volume, particularly without adequate rest and recovery, can have detrimental effects and may paradoxically increase ER stress, as observed in overtraining scenarios (Marafon et al., 2022; Pereira et al., 2016). The biphasic response of ER stress to exercise highlights the necessity of meticulously evaluating exercise prescription parameters, including intensity, duration, frequency, and rest periods, to achieve beneficial adaptations in ER health within skeletal muscle.

**Figure 3:**
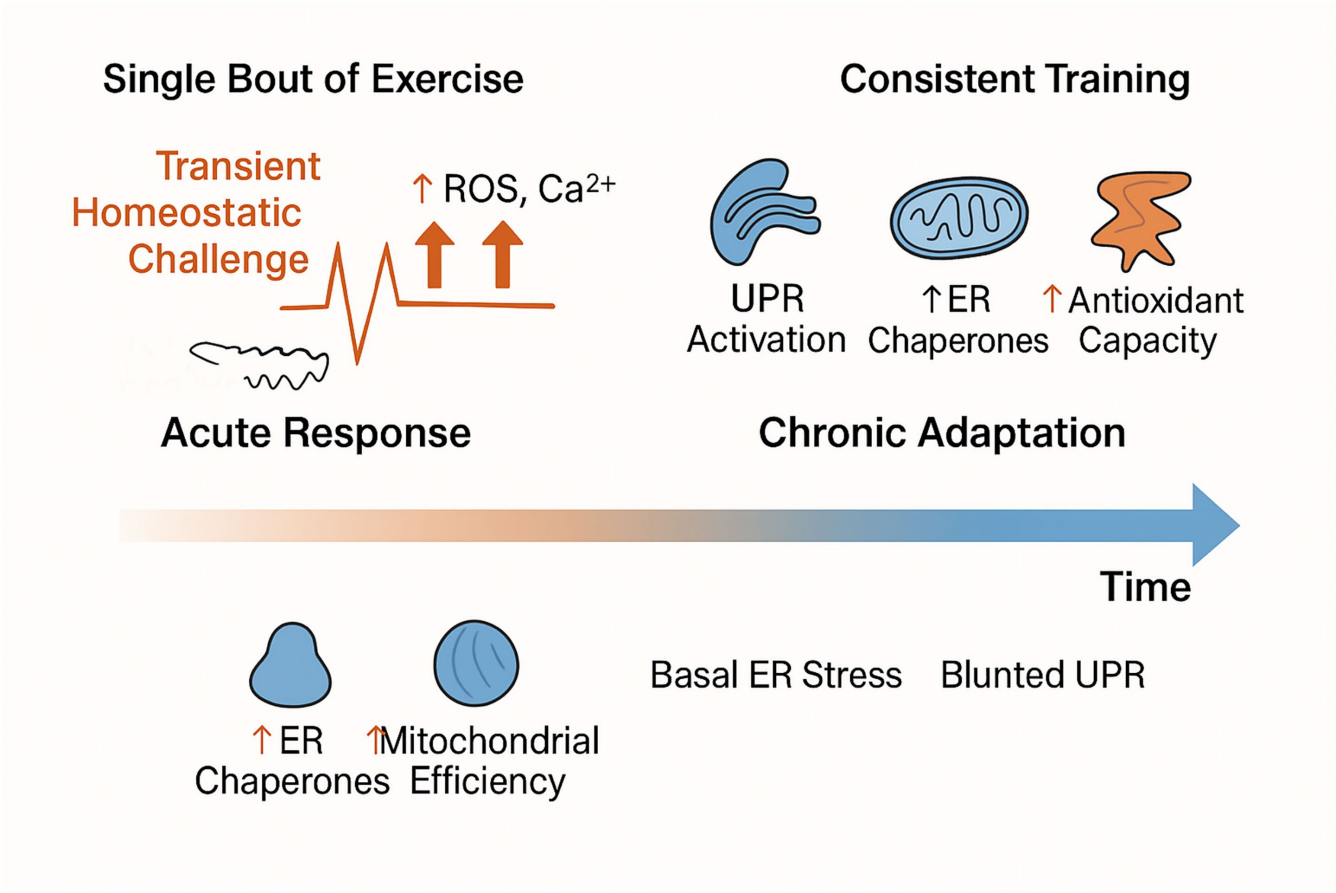
Acute versus chronic adaptations of exercise-induced ER stress responses. A single bout of exercise acts as a transient homeostatic challenge, increasing ROS and calcium flux, which triggers mild and short-lived UPR activation, upregulating protective chaperones and antioxidant enzymes (acute response). With consistent training, these repeated signals drive long-term adaptations, including elevated baseline ER chaperones, improved mitochondrial efficiency, and enhanced antioxidant capacity. Trained muscle thus exhibits lower basal ER stress and a blunted, more efficient UPR under stress, reflecting a resilient cellular phenotype.

This distinction between the acute response and chronic adaptation can be visualized as a timeline (Figure 3). A single bout of exercise acts as a transient homeostatic challenge, causing a rapid increase in ROS and calcium flux that triggers a mild, short-lived UPR activation. This is a crucial signaling event that results in the temporary upregulation of protective chaperones and antioxidant enzymes. With consistent training over weeks and months, these repeated acute signals drive a long-term adaptive state. This chronic adaptation is characterized by an increased baseline pool of ER chaperones, greater mitochondrial efficiency, and enhanced antioxidant capacity (Hetz and Papa, 2018; Silva et al., 2022; Farrell and Turgeon, 2021). Consequently, the trained muscle exhibits lower basal ER stress and a blunted, more efficient UPR response when faced with subsequent stressors, signifying a more resilient cellular phenotype.

### 4.2 Intensity and type of exercise

ER stress in skeletal muscle has been shown to be induced by both endurance (aerobic) and resistance exercise, although the specific markers and the extent of the response may vary depending on the type of exercise and the intensity of the exercise (Wang et al., 2011; Koulmann and Bigard, 2006). HIIT is emerged as a potentially beneficial exercise modality, with some studies indicating that it can lead to a decrease in ER stress markers within skeletal muscle (Kim et al., 2014; Liu et al., 2013). This finding challenges the notion that only moderate-intensity exercise is beneficial and suggests that higher intensity exercise, when appropriately managed, could trigger distinct adaptive pathways in the ER.

Regular moderate-intensity exercise appears to be generally beneficial for attenuating basal ER stress levels in skeletal muscle, promoting a more resilient cellular environment (de Sousa Fernandes et al., 2023; El Assar et al., 2022). In contrast, low-intensity exercise may be inadequate to produce notable alterations in ER stress markers, especially when conducted in a fasted condition (Marafon et al., 2022; Lee et al., 2015). Furthermore, it is important to consider that the response to exercise-induced ER stress can differ depending on the specific muscle being examined (de Sousa Fernandes et al., 2023; Kim et al., 2010; Kim et al., 2018). For instance, the soleus and EDL muscles may express different ER stress markers after the same exercise. This heterogeneity highlights the complexity of skeletal muscle and the need for tissue-specific considerations when designing exercise interventions aimed at modulating ER stress.

### 4.3 Molecular mechanisms of exercise-induced ER stress modulation

Several molecular mechanisms may explain how exercise modulates ER stress in skeletal muscle. PGC-1α, a key regulator of mitochondrial biogenesis and energy metabolism in muscle, is crucial for mediating exercise-induced adaptations (Afroze and Kumar, 2019; Baar, 2004; Feng et al., 2013; Lira et al., 2010). Research indicates that PGC-1α interacts with cleaved ATF6α, a transcription factor activated during the unfolded protein response (UPR), to promote an adaptive UPR in skeletal muscle post-exercise (Afroze and Kumar, 2019; Wu et al., 2011; Jung and Kim, 2014). This interaction indicates that PGC-1α is essential for ER stress adaptation in muscle, potentially improving ER health and resilience.

**Figure 4:**
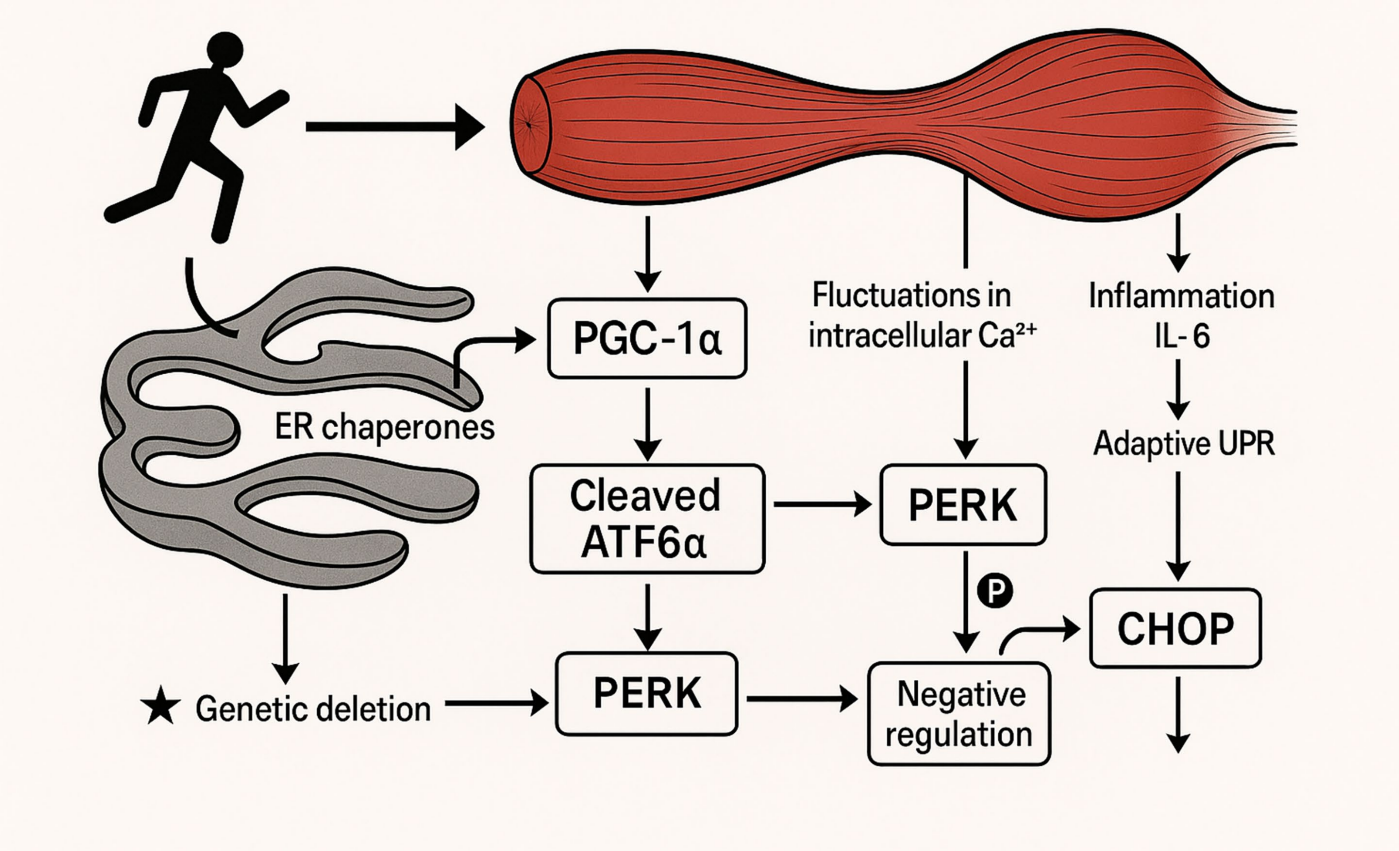
This diagram illustrates how exercise influences ER stress pathways in skeletal muscle. Key components include the activation of PGC-1α, which interacts with cleaved ATF6α to promote an adaptive UPR. Exercise also increases ER chaperones and induces fluctuations in intracellular calcium (Ca^2+^), which can trigger PERK pathway activation. While the PERK/eIF2α/CHOP axis may exert negative regulation, deletion of CHOP improves muscle adaptation. Additionally, IL-6-mediated inflammation contributes to ER stress modulation, highlighting the complexity and specificity of UPR pathway roles in exercise adaptation.

Exercise also increases the expression of ER chaperones, proteins that help protein folding and restore ER homeostasis, reducing ER stress (Deldicque, 2013). Interestingly, different UPR arms may play different roles in exercise adaptation. PERK/eIF2α/CHOP pathway may negatively regulate muscle homeostasis after exercise training, while the ATF6α pathway appears to be involved in adaptive response and recovery from exercise-induced damage (Afroze and Kumar, 2019; Ogborn, 2013; Chen et al., 2023; Vargas-Mendoza et al., 2019). For example, it is demonstrated that the deletion of CHOP genetic material leads to an enhancement of exercise adaptation in mice with skeletal muscle-specific PGC-1α knockout (Afroze and Kumar, 2019; Kristensen et al., 2018). Intracellular calcium level fluctuations during muscle contraction in exercise are considered a potential trigger for UPR activation (Afroze and Kumar, 2019; Ito et al., 2018; Kano et al., 2012). Additionally, Inflammation, especially with respect to the cytokine IL-6, may contribute to the intricate regulation of ER stress homeostasis during exercise (Marafon et al., 2022; Ropelle et al., 2010). The opposing roles of different UPR arms in exercise-induced muscle adaptation underscore the complexity of this cellular response and the need for targeted interventions that selectively modulate specific UPR pathways to maximize benefits (Figure 4). Table 2 summarizes what exercise does for ER stress in neurodegenerative diseases.

**Table 2:** ER stress markers in skeletal muscle (rodents) after exercise interventions.

Marker	Muscle	Exercise protocol	Effect	References
BiP	Soleus	Aerobic exercise	Decreased	de Sousa Fernandes et al. (2023)
BiP	EDL	Aerobic exercise	Decreased	de Sousa Fernandes et al. (2023)
CHOP	Gastrocnemius	Aerobic exercise (8 weeks)	Decreased	de Sousa Fernandes et al. (2023)
ATF4	Quadriceps	Aerobic exercise (4 weeks)	Increased	de Sousa Fernandes et al. (2023)
BiP	Quadriceps	Aerobic exercise (4 weeks)	Increased	de Sousa Fernandes et al. (2023)
XBP1s	Quadriceps	Aerobic exercise (4 weeks)	Increased	de Sousa Fernandes et al. (2023)
p-PERK	Heart	Swimming	Decreased	de Sousa Fernandes et al. (2023)
BiP	Heart	High-intensity training (5 weeks)	Decreased	Kim et al. (2014)
ATF4	Heart	High-intensity training (5 weeks)	Decreased	Kim et al. (2014)
CHOP	Heart	High-intensity training (5 weeks)	Decreased	Kim et al. (2014)
BiP	Heart	Low-intensity training (5 weeks)	No change	Kim et al. (2014)
ATF4	Heart	Low-intensity training (5 weeks)	Increased	Kim et al. (2014)
CHOP	Heart	Low-intensity training (5 weeks)	No change	Kim et al. (2014)
BiP	Skeletal muscle	High-intensity training (5 weeks)	Decreased	Kim et al. (2014)
ATF4	Skeletal muscle	High-intensity training (5 weeks)	Decreased	Kim et al. (2014)
CHOP	Skeletal muscle	High-intensity training (5 weeks)	Decreased	Kim et al. (2014)
BiP	Skeletal muscle	Low-intensity training (5 weeks)	No change	Kim et al. (2014)
ATF3	Skeletal muscle	Low-intensity training (5 weeks)	Increased	Kim et al. (2014)
ATF4	Skeletal muscle	Low-intensity training (5 weeks)	No change	Kim et al. (2014)

### 4.4 The interconnected stress web: UPR, mitochondria, oxidative stress, and inflammation

The progression of muscle decline in neurodegenerative diseases is not driven by a single pathway but by a destructive interplay between several cellular stress responses. The UPR, mitochondrial dysfunction, oxidative stress, and inflammation form a self-perpetuating ‘vicious cycle.’ Understanding how exercise modulates ER stress requires appreciating its ability to intervene at multiple points within this interconnected web.

In a pathological state, these four elements amplify one another. Mitochondrial dysfunction is a central node; damaged mitochondria produce less ATP and leak excessive reactive oxygen species (ROS), causing oxidative stress. This surge in ROS directly damages lipids and proteins within the ER, impairing calcium handling and protein folding, which in turn triggers the UPR (Misrani et al., 2021; Kowalczyk et al., 2021). A chronically activated UPR then feeds back to harm the mitochondria. It disrupts the critical physical and functional connection at the mitochondria-associated membranes (MAMs), impairing calcium transfer needed for bioenergetics and activating mitochondria-mediated apoptosis through effectors like CHOP. Furthermore, the UPR, particularly via the IRE1α and PERK pathways, activates pro-inflammatory signaling cascades like NF-κB and JNK (Senft and Ronai, 2015; Bravo et al., 2012). The resulting chronic inflammation closes the loop by generating more ROS and directly impairing mitochondrial function, thus perpetuating a cycle of cellular damage, energy crisis, and muscle wasting.

Exercise is uniquely positioned to break this vicious cycle and foster a ‘virtuous cycle’ of cellular health.

- *Mitochondrial Quality Control:* Regular exercise is the most powerful physiological stimulus for *mitochondrial biogenesis*, driven by the master regulator PGC-1α. This process not only creates new, healthy mitochondria but also stimulates mitophagy, the selective removal of damaged ones. The result is a healthier mitochondrial pool that produces ATP efficiently with minimal ROS leakage, directly combatting the cycle’s origin (Memme and Hood, 2020; Memme et al., 2021; Kyriazis et al., 2022).
- *Antioxidant Defenses:* Chronic exercise upregulates the body’s endogenous antioxidant systems. It activates the Nrf2 transcription factor, which increases the expression of key antioxidant enzymes like superoxide dismutase (SOD) and catalase. This enhanced defensive capacity allows the muscle to better neutralize ROS, protecting the ER and other organelles from oxidative damage (Jomova et al., 2023; de Lemos et al., 2012).
- *Adaptive UPR Preconditioning:* As previously discussed, acute exercise induces a transient, physiological UPR. This response is not pathological but adaptive; it increases the expression of ER chaperones and enhances the ER’s folding capacity. This ‘preconditioning’ makes the ER more resilient to subsequent, more severe stressors, effectively raising the threshold for triggering a maladaptive UPR (Salminen et al., 2020).
- *Systemic Anti-Inflammatory Effects:* While acute exercise is pro-inflammatory, regular training has potent *anti-inflammatory effects*. It reduces chronic low-grade inflammation by decreasing visceral fat mass (a major source of inflammatory cytokines) and promoting the release of anti-inflammatory myokines from the muscle. This dampens the inflammatory signaling that would otherwise contribute to oxidative stress and mitochondrial damage (Flynn et al., 2007).

## 5 Exercise interventions and their effects on ER stress and muscle function in neurodegenerative diseases

While the primary focus of this review is on skeletal muscle, a comprehensive understanding of exercise’s therapeutic benefits requires acknowledging its profound effects on ER stress within the brain itself. Exercise combats neuronal ER stress through several interconnected mechanisms. First, it enhances cerebral proteostasis by upregulating the expression of molecular chaperones, such as heat shock proteins (HSPs), which improves the brain’s capacity to correctly fold proteins and clear toxic aggregates like amyloid-*β* and tau (Wankhede et al., 2022; Xu et al., 2022; Hu et al., 2022). Second, exercise robustly increases levels of Brain-Derived Neurotrophic Factor (BDNF), a critical neurotrophin that promotes neuronal survival and resilience against stressors, including ER stress-induced apoptosis (Wang and Holsinger, 2018). Third, physical activity exerts potent anti-inflammatory and antioxidant effects, reducing neuroinflammation and oxidative damage, both of which are primary triggers of the UPR in neurons (Simioni et al., 2018; Wang et al., 2023). Finally, by improving cerebral blood flow and promoting autophagy, exercise facilitates the efficient clearance of misfolded proteins, thereby alleviating the initial burden that would otherwise trigger an ER stress response (Gao et al., 2025; Li et al., 2024). These neuroprotective actions, combined with the peripheral effects on muscle, underscore the holistic therapeutic potential of exercise in combating neurodegenerative diseases.

### 5.1 Parkinson’s disease

According to the available evidence, individuals who suffer from PD may reap significant benefits from engaging in physical activity, particularly aerobic exercise and resistance training (Falvo et al., 2008; Schootemeijer et al., 2020). Regular physical activity improves motor function, reduces bradykinesia, and enhances balance and coordination, and ultimately improve the overall quality of life for PD patients (Duranti and Villa, 2024; Borrione et al., 2014). Exercise may benefit PD muscle function by improving muscle strength, mitochondrial function, and systemic inflammation (Duranti and Villa, 2024; Kelly et al., 2014). The findings indicate a beneficial effect of exercise on motor symptoms in PD; however, the specific influence of exercise interventions on ER stress markers in the muscle tissue of Parkinson’s patients or animal models is not clearly addressed in the provided excerpts. Considering the established function of ER stress in muscle dysfunction and the capacity of exercise to influence ER stress in healthy muscle, future research should explore this relationship to enhance understanding of the mechanisms that underlie the benefits of exercise in PD and to potentially refine exercise interventions for this demographic.

### 5.2 Alzheimer’s disease

Exercise training has demonstrated potential in mitigating cerebrovascular dysfunction associated with AD by influencing ER stress within the brain. Research indicates that physical activity can diminish the expression of abnormal ER stress markers in the brains of animal models of AD, specifically p-IRE1α, CHOP, and p-eIF2α (Hong et al., 2020; Lange-Asschenfeldt and Kojda, 2008; Medinas et al., 2021). This suggests that exercise can modulate ER stress-dependent endothelial dysfunction, which is linked to AD. Evidence suggests that exercise can improve cerebral blood flow, reducing amyloid-*β* accumulation, neuronal cell death, and cognitive decline in AD patients (Ribarič, 2022; Tan et al., 2021). While these findings highlight the neuroprotective effects of exercise in AD, research specifically examining the effects of exercise on ER stress within muscle tissue of AD models or patients and its potential contribution to improved physical function is less prominent in the provided snippets. Considering the possible association between ER stress and muscle atrophy in AD, further investigation into whether exercise also alleviates ER stress in muscle tissue in this context could provide a more complete understanding of its therapeutic benefits for both cognitive and physical function.

### 5.3 Huntington’s disease

The research snippets provided do not specifically examine the effects of exercise interventions on ER stress and muscle function in models or patients with HD. However, one snippet (Simha et al., 2025) mentions a case study where an Ayurvedic treatment regimen, including *Sida cordifolia* (SC), led to improvements in motor symptoms and a decrease in ER stress in a model of HD (Simha et al., 2023; Tung et al., 2025). This suggests that interventions targeting ER stress might have the potential to improve motor function in HD. Given the contribution of ER stress in HD neuronal pathology and the evidence for mHTT expression in peripheral tissues, including muscle, more research on the effects of exercise on ER stress and muscle function is needed. Such studies may help manage this disease’s debilitating motor symptoms.

The lack of evidence for exercise interventions in HD is also due to significant practical challenges. The characteristic motor symptoms of HD, such as chorea (involuntary movements) and dystonia, make the implementation and standardization of exercise protocols extremely difficult. This creates barriers to conducting the rigorous clinical trials needed to assess both functional outcomes and underlying molecular changes like ER stress.

### 5.4 Amyotrophic lateral sclerosis

**Figure 5:**
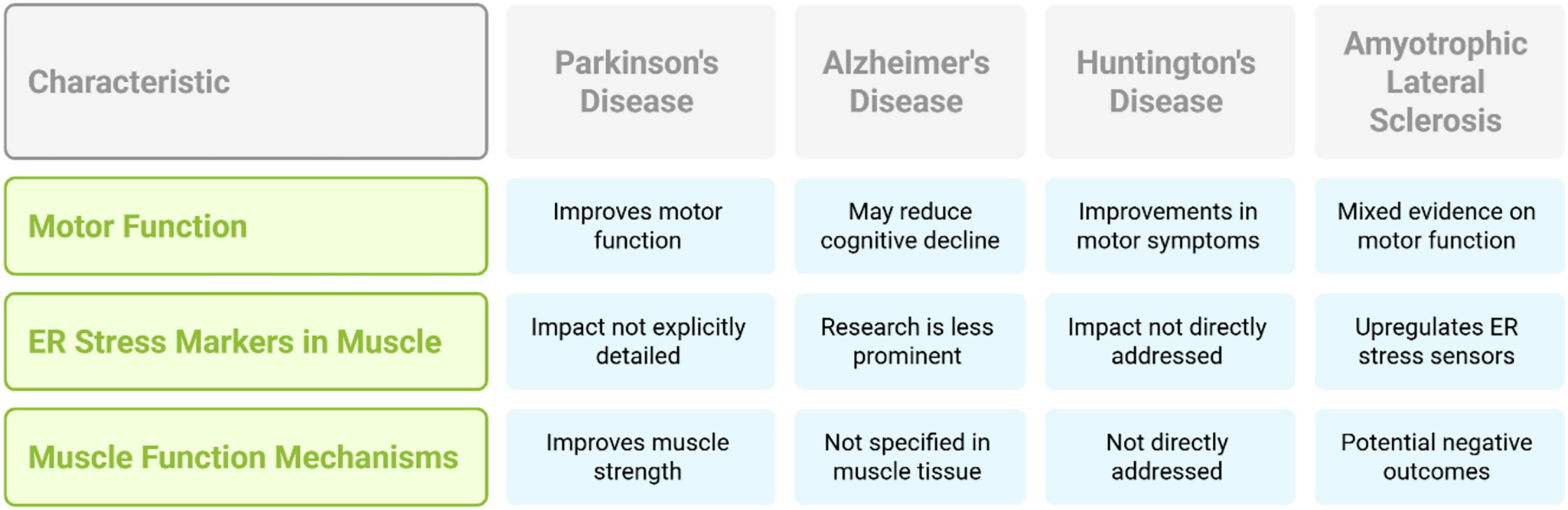
Exercise effects on ER stress and muscle function.

The impact of exercise in ALS is multifaceted and has been a topic of ongoing discussion (Chen et al., 2008). Some studies indicate that exercise may enhance the quality of life for individuals with ALS, others indicate potential negative outcomes, particularly with strenuous exercise, possibly due to increased metabolic demands on already compromised motor neurons and muscle (Duranti and Villa, 2023; de Almeida et al., 2012). Snippet (Chen et al., 2015) mentions that exercise is proved to upregulate ER stress sensors and ER chaperones in skeletal muscle, but it does not specify whether this occurs in the context of ALS. Snippets (Deldicque, 2013; Gallot and Bohnert, 2021) discuss how exercise affects muscle ER stress, which might be relevant to understanding the potential impact in ALS. However, there is a lack of direct evidence within the provided snippets regarding whether exercise interventions specifically modulate ER stress markers in the muscle tissue of ALS models or patients. The conflicting evidence surrounding exercise in ALS underscores the critical need for highly individualized and carefully monitored exercise programs for individuals with this condition. Future research should aim to elucidate how different exercise regimens affect ER stress in muscle in ALS, which could help in developing safer and more effective exercise guidelines for this vulnerable population (Tables 1, 2, Figure 5).

### 5.5 Caveats and controversies: the double-edged sword of exercise

While this review highlights the therapeutic potential of exercise, it is crucial to acknowledge that its effects are not universally positive and the literature contains significant contradictory evidence, particularly for ALS. The concept of hormesis is central: while moderate stress can be adaptive, excessive stress can be damaging, and this balance is especially delicate in neurodegenerative diseases.

The controversy is most pronounced in ALS, where some studies have even suggested a link between elite athletic activity and an increased risk of developing the disease (Hu and Robertson, 2020; Chapman et al., 2023). Furthermore, preclinical studies in SOD1 mutant mouse models have shown conflicting results based on exercise intensity. While moderate, voluntary wheel running has sometimes shown modest benefits or no effect, forced, high-intensity exercise (such as forced treadmill running or swimming) has been demonstrated to accelerate disease onset, worsen motor neuron loss, and shorten lifespan (Hu and Robertson, 2020; Scaricamazza et al., 2024; Manzanares et al., 2018). These negative outcomes are thought to result from exacerbating the underlying pathology, including overwhelming cellular bioenergetics and increasing oxidative stress on already vulnerable motor neurons.

This “double-edged sword” concept, while most stark in ALS, is relevant across all neurodegenerative diseases. In advanced stages of PD or AD, for instance, high-intensity exercise could increase the risk of falls, injury, or adverse cardiovascular events (Berg and Cassells, 1992; Ernst et al., 2023). Therefore, the key takeaway is that the intensity, duration, and type of exercise are critical variables that determine whether the outcome is beneficial or detrimental. Future research must move beyond simply asking *if* exercise is helpful and focus on defining precise, safe, and effective exercise prescriptions tailored to the specific disease, its stage, and the individual patient’s capacity.

## 6 Conclusion and future directions

In conclusion, Endoplasmic Reticulum (ER) stress is a significant contributor to the pathogenesis of Parkinson’s Disease (PD), Alzheimer’s Disease (AD), Huntington’s Disease (HD), and Amyotrophic Lateral Sclerosis (ALS), affecting both neuronal function and skeletal muscle integrity. Exercise has emerged as a promising, non-pharmacological modulator of ER stress. Chronic, moderate-intensity training generally attenuates ER stress responses, while acute exercise can induce a transient, adaptive stress response. The type and intensity of exercise appear to be critical variables, with modalities like high-intensity interval training showing potential benefits.

However, the existing evidence on how exercise interventions impact ER stress and muscle function in specific neurodegenerative diseases is still developing. While exercise clearly benefits motor function in PD and may reduce brain ER stress in AD, its direct effects on muscle tissue in these conditions require more research. Evidence for exercise in HD is particularly scarce, and its role in ALS remains complex and controversial, necessitating highly individualized and cautious approaches. The intricate crosstalk between ER stress, mitochondrial dysfunction, inflammation, and protein aggregation suggests that the most effective therapeutic strategies will need to target these multiple interconnected pathways.

### 6.1 Critical context and limitations of the current field

To properly contextualize these findings, it’s important to frame the therapeutic potential of exercise within the current pharmacological landscape. While the ER stress markers discussed are clear pathological contributors, there are currently no FDA-approved drugs that directly target these core Unfolded Protein Response (UPR) pathways for treating neurodegenerative diseases. Research into pharmacological interventions is ongoing, with promising strategies like chemical chaperones (e.g., TUDCA and sodium phenylbutyrate) remaining largely investigational (Ioannou et al., 2025). This therapeutic gap underscores the immense value of non-pharmacological approaches like exercise, which offers a readily available strategy to enhance cellular resilience.

Furthermore, it is critical to address the limitations of extrapolating findings from preclinical rodent models to human conditions. Animal models often fail to capture the complexity of sporadic human diseases, which arise from a mix of genetic and environmental factors. The accelerated disease progression in a short-lived animal does not fully mimic the decades-long development in humans (Alexandra Lopes and Guil-Guerrero, 2025). Significant physiological and metabolic differences mean that the dose, type, and cellular response to an exercise intervention in a mouse cannot be directly translated to a human patient. Therefore, while animal models provide a strong rationale for exercise, their findings must be interpreted with caution.

Finally, future research must carefully consider several potential confounders that influence the relationship between exercise and ER stress. A patient’s diet, for instance, can independently modulate ER stress (Singh et al., 2024; Stults-Kolehmainen and Sinha, 2014). The presence of comorbidities like obesity or type 2 diabetes can create a high basal stress level that may blunt the benefits of exercise (Colberg et al., 2010; Zahalka et al., 2019). Moreover, sex differences and an individual’s lifelong training history are critical factors that can alter the cellular response to an exercise bout. Future clinical trials must be designed to control for these variables to develop truly personalized exercise prescriptions.

### 6.2 A roadmap for future research and application

#### 6.2.1 Translational considerations and potential biomarkers

A major hurdle in translating preclinical findings is the reliance on invasive muscle biopsies. To facilitate clinical trials, the development of less invasive biomarkers is essential. Potential biomarkers could be tiered based on feasibility and directness:

- Tier 1 (Direct/Invasive): Muscle Biopsies remain the gold standard. Key markers to quantify include the phosphorylation of PERK and eIF2α, mRNA levels of spliced XBP1, and protein levels of adaptive (BiP/GRP78) versus pro-apoptotic (CHOP) markers (Walter et al., 2015; Rozpedek et al., 2016).
- Tier 2 (Less-Invasive/Circulating): Blood-Based Markers offer a more practical alternative. This includes measuring secreted ER chaperones like GRP78/BiP in plasma, though their tissue origin can be ambiguous (Lee, 2005). Other promising candidates include myokines regulated by ER stress, such as Fibroblast Growth Factor 21 (FGF21), and specific circulating microRNAs released from stressed muscle (Lee, 2005; Cicek et al., 2024).
- Tier 3 (Functional/Imaging): Non-invasive Proxies can provide indirect evidence of improved cellular health. For example, ^31^P-magnetic resonance spectroscopy (^31^P-MRS) can assess mitochondrial function, while advanced ultrasound techniques can measure muscle quality changes that reflect improvements in cellular composition (Bendahan et al., 2006).

#### 6.2.2 Integrating omics and advanced clinical trial designs

Future research should incorporate omics-based findings. High-throughput technologies like transcriptomics, proteomics, and metabolomics can provide an unbiased, systems-level view of the molecular adaptations to exercise. For instance, RNA-sequencing of muscle biopsies from patients in an exercise trial could reveal entire networks of genes related to the UPR and mitochondrial biogenesis, providing a comprehensive signature of exercise’s benefits.

Furthermore, there is a need to enhance the design of clinical trial evidence. Historically, most exercise trials have focused on functional outcomes. A critical future direction is the design of trials that pair these clinical measures with the systematic collection of biological samples for molecular analysis. Measuring ER stress markers as secondary outcomes in human trials is essential to finally link the proposed mechanism to the clinical benefit.

#### 6.2.3 Proposed experimental designs to address current gaps

To move the field forward, research must adopt more sophisticated experimental designs. Preclinically, studies should shift towards models that better mimic the human condition, such as using voluntary exercise in aged or late-stage disease models. A powerful design would be a longitudinal study combining multi-modal exercise with intermittent muscle biopsies for single-cell transcriptomics to understand how different muscle fiber types adapt over time.

Clinically, the next step is to implement longitudinal, multi-arm intervention trials. A robust design would involve recruiting a well-defined patient cohort (e.g., early-stage PD) and randomizing them into groups: (1) a control group, (2) a moderate-intensity continuous training group, and (3) a high-intensity interval training (HIIT) group. The intervention would last for at least 6 months, with multi-layered outcomes including (a) functional measures (e.g., 6-min walk test), (b) invasive molecular measures (muscle biopsies), and (c) non-invasive biomarkers.

#### 6.2.4 Framework for implementing individualized exercise prescriptions

Moving from principle to practice requires a clear framework for individualizing exercise. A multidisciplinary approach is essential and should include:

1. *Comprehensive Baseline Assessment:* Each patient must undergo a thorough evaluation by a team including a physician and a physical therapist to assess disease stage, functional capacity, comorbidities, and the patient’s personal goals.
2. *Prescription Based on “Start Low, Go Slow”:* The initial prescription should be conservative. Intensity can be guided by the *Rating of Perceived Exertion (RPE) scale* and heart rate monitoring, with the program progressively titrated based on tolerance.
3. *Integration of Wearable Technology:* Modern wearables can provide real-world data on activity, sleep, and heart rate variability, allowing for remote monitoring and patient self-management.
4. *Future Biomarker-Guided Personalization:* The ultimate goal of “precision exercise medicine” is to create a feedback loop where a patient’s exercise prescription is adjusted not just based on function, but also on how their molecular biomarkers respond to the training, ensuring the dose is optimized to promote an adaptive cellular response.
